# Polysomnographic Evaluation of Sleep Bruxism Intensity and Sleep Architecture in Nonapneic Hypertensives: A Prospective, Observational Study

**DOI:** 10.3390/jcm11113113

**Published:** 2022-05-31

**Authors:** Justyna Kanclerska, Mieszko Wieckiewicz, Rafal Poreba, Anna Szymanska-Chabowska, Pawel Gac, Anna Wojakowska, Weronika Frosztega, Monika Michalek-Zrabkowska, Grzegorz Mazur, Helena Martynowicz

**Affiliations:** 1 Department and Clinic of Internal Medicine, Occupational Diseases, Hypertension and Clinical Oncology, Wroclaw Medical University, 50-556 Wroclaw, Poland; kanclerska.justyna@gmail.com (J.K.); rafal.poreba@umw.edu.pl (R.P.); aszyman@mp.pl (A.S.-C.); anna.wojakowska@umw.edu.pl (A.W.); weronika.frosztega@gmail.com (W.F.); monika.michalek1@gmail.com (M.M.-Z.); grzegorz.mazur@umw.edu.pl (G.M.); 2 Department of Experimental Dentistry, Wroclaw Medical University, 26 Krakowska St., 50-425 Wroclaw, Poland; m.wieckiewicz@onet.pl; 3 Department of Population Health, Division of Environmental Health and Occupational Medicine, Wroclaw Medical University, Mikulicza-Radeckiego 7, 50-368 Wroclaw, Poland; pawel.gac@umw.edu.pl

**Keywords:** arterial hypertension, sleep bruxism, sleep architecture, snoring, polysomnography

## Abstract

Sleep bruxism (SB) is a repetitive jaw muscle activity characterized by clenching or grinding of the teeth, which is classified under sleep-related movement disorders in the International Classification of Sleep Disorders—Third Edition. Because the potential common pathomechanism of SB and arterial hypertension is the activation of the sympathetic system as well as an increase in inflammatory factors, we aimed to examine the intensity of SB and the sleep architecture among patients with arterial hypertension. The study included a total of 91 Caucasian adult patients, among whom 31 had arterial hypertension diagnosed according to the current European Society of Cardiology/European Society of Hypertension (ESC/EHS) hypertension guidelines. The control group consisted of 61 normotensive patients. Patients with obstructive sleep apnea were excluded. A single full-night polysomnographic examination was conducted in the Sleep Laboratory, and then the results were analyzed based on the guidelines of the American Academy of Sleep Medicine. Bruxism episode index (BEI) was higher in the hypertensive group compared to normotensives. The groups also showed statistically significant differences in polysomnographic sleep indexes. Similar to BEI, arousal index, apnea–hypopnea index, and snoring were higher in hypertensives compared to normotensives. On the other hand, the mean and minimal oxygen saturation were lower in hypertensives compared to normotensives. A statistically significant positive correlation was observed between oxygen desaturation index and BEI in the hypertensive group, whereas this correlation was not statistically significant in the case of normotensives. In summary, nonapneic hypertensives had higher SB intensity, altered sleep architecture, decreased mean oxygen saturation, and increased snoring compared to normotensives. The results suggest that dental screening is necessary for patients with arterial hypertension, especially those presenting with the symptoms of SB.

## 1. Introduction

The definition and classification of bruxism have varied throughout the years. In 2013, Lobezzo first described bruxism as “a repetitive jaw muscle activity characterized by clenching or grinding of the teeth and/or bracing or thrusting of the mandible” [1]. A person can experience bruxism either while sleeping (sleep bruxism (SB)) or while staying awake (awake bruxism (AB)) [2]. Currently, SB is classified as a sleep-related movement disorder in the International Classification of Sleep Disorders—Third Edition, whereas earlier it was reported by the American Academy of Sleep Medicine (AASM) as parasomnia [3,4].

In 2018, an international bruxism consensus was published that clarified the previous definition along with further findings. The following definition clearly distinguishes SB from AB: “Sleep bruxism is a masticatory muscle activity during sleep that is characterized as rhythmic (phasic) or non-rhythmic (tonic) and is not a movement disorder or a sleep disorder in otherwise healthy individuals” [5]. It was stated that bruxism should not be regarded as a disorder, but rather a behavior that can be a risk (and/or protective) factor for specific clinical outcomes.

According to the latest research, SB is regulated centrally (pathophysiological, neurological, and psychosocial factors) and not peripherally (morphological and local factors) [6]. One of the key pathomechanisms of bruxism is the activation of the sympathetic system, which is also the main cause of arterial hypertension [7,8]. It has been shown that bruxism is accompanied by disturbances in autonomic activity, especially sympathetic vasoconstrictor dysfunction [9,10]. Increased levels of stress and anxiety, which are the major determinants of SB, can modulate sympathetic activity [11]. Furthermore, SB episodes can occur along with cortical arousal which can be observed during electroencephalography, as well as tachycardia, in association with transient elevations of sympathetic tone [12]. This implies the involvement of neurotransmission pathways (e.g., serotonin and dopamine pathways) in the pathogenesis of SB [13].

Arterial hypertension is a prevalent condition that can increase the risk of heart, brain, kidney, and other diseases. It shares common pathophysiology with various sleep disturbances, such as obstructive sleep apnea (OSA) [14], restless leg syndrome [15], and insomnia [16]. Several studies have linked the presence of sleep disorders with hypertension [17]. For instance, a recent work by Michalek-Zrabkowska et al. revealed that severe bruxers (with bruxism episode index (BEI) > 4/h) exhibited higher systolic blood pressure variability during sleep compared to individuals with BEI ≤ 4/h [11]. It was found that increased BEI is an independent risk factor for hypertension [18]. In addition, Nashed et al. pointed out that blood pressure fluctuations during sleep are associated with rhythmic masticatory muscle activity (RMMA) [19].

The significance of dopamine and serotonin [20] gene alterations in SB was investigated in some studies, and the results indicated a change in central dopamine [21] activity mostly among bruxers as well as in patients with restless leg syndrome [22]. As proven by Wieckiewicz et al., the etiology of SB may involve potential variations in the genes encoding for serotonin receptors (HTR2A) and dopamine receptors (DRD1). It seems that the HTR2A gene more likely influences the pathogenesis of SB, and its polymorphism may link SB with OSA.

Data on the relationship between arterial hypertension and SB are scarce. Moreover, the literature contains no studies based on polysomnography, which is a gold standard in the diagnosis of SB. Due to the fact that SB and arterial hypertension share the same pathomechanism, at least partially, we aimed to evaluate the intensity of SB and the sleep architecture among patients with arterial hypertension. In recent years, particular attention has been paid to the study of sleep architecture in arterial hypertension, as it has been proven that successive micro-arousals increase cardiovascular risk. Our study aims to bring attention to the patients with hypertension that could also affect the intensification of SB taking the common pathomechanism into consideration.

The null hypothesis of the study was that hypertensives had increased SB intensity and altered sleep architecture. The scope of the study was to determine whether nonapneic patients with hypertension have altered sleep structures and different severity levels of bruxism compared to normotensives.

## 2. Materials and Methods

### 2.1. Standard Protocol Approvals, Registrations, and Patient Consents

**Figure 1:**
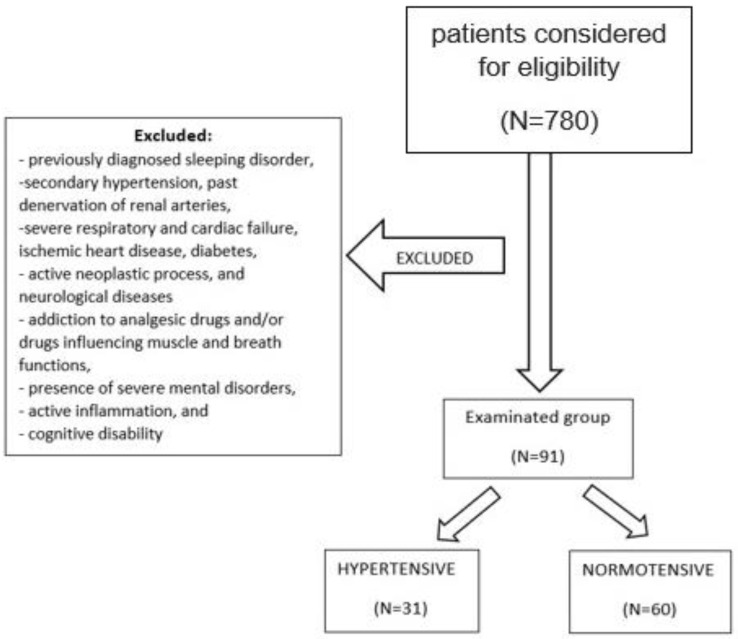
Flowchart.

The prospective, observational study included a total of 91 Caucasian adults (Table 1). Among them, 31 were diagnosed with arterial hypertension in the medical interview, based on the ESC/EHS hypertension guidelines [23]. The control group consisted of normotensive individuals (*n* = 60) who had no medical history of hypertension and did not use antihypertensive medications; their blood pressure on admission was <140/90 mmHg. A sample size calculator was used to determine sample size. The calculator’s standard assumptions for our population were used: population size 3,000,000, fraction size 0.3, confidence level 0.95, and a maximum error of 10%. The required group size was 81. We recruited a group of 91 people, which is larger than required. The examination was carried out in the Sleep Laboratory of the Department and Clinic of Internal Medicine, Occupational Diseases, Hypertension, and Clinical Oncology at the Wroclaw Medical University, Poland. The study was approved by the Ethical Committee of Wroclaw Medical University (ID KB-407/2022). The trial was not registered in a WHO directory. The inclusion criteria were age over 18 years and willingness to provide written consent for the examination. The exclusion criteria were previously diagnosed sleeping disorder, secondary hypertension, past denervation of renal arteries, apnea–hypopnea index (AHI) > 10 in polysomnography, severe respiratory and cardiac failure, ischemic heart disease, diabetes, active neoplastic process, and neurological diseases as well as treatment with or addiction to analgesic drugs and/or drugs influencing muscle and breath functions, presence of severe mental disorders, active inflammation, and cognitive disability (Figure 1).

### 2.2. Study Design and Subjects

The study group included 63 females and 28 males, of which 31 had hypertension. Among patients with hypertension, 14 were males and 17 were females (Table 1). A single full-night polysomnographic examination was conducted among the patients in the Sleep Laboratory. The bioelectrical function of the brain was recorded by electroencephalography, eye movements by electrooculography, muscular tension from tibial electrodes by electromyography (EMG), air flow from the nasal pressure sensor, and chest and abdomen movements by inductive plethysmography. In addition, blood saturation was measured by pulse oximetry, and heart function was assessed by electrocardiography. The body position of the patients was also studied. The presence of SB was analyzed by bilateral masseter EMG, and the audio and video recordings of bruxism episodes were scored according to the AASM standards in three forms: phasic, tonic, and mixed. While considering SB, it was assumed that the EMG bursts of the same episode should not be separated by > 3 s, and the EMG activity had to be at least twice the amplitude of the background EMG [19]. A qualified physician (H.M.) from the Sleep Laboratory of the Wroclaw Medical University, Poland scored and manually analyzed the collected data in accordance with the AASM guidelines using the Noxturnal system Nox-A1(Nox Medical, Reykjavik, Iceland, version 5.1).

### 2.3. Data Analysis

Statistical analyses were performed using the Dell Statistica 13.1 application (Dell Inc., Round Rock, TX, USA). Arithmetic means and standard deviations were calculated for quantitative variables, and percentages were calculated for qualitative variables. The distribution of variables was verified using the Shapiro–Wilk test. The hypotheses were tested with t-tests dedicated to unrelated and related variables, respectively. The differences between the mean values at a *p*-value of <0.05, <0.01, and <0.001 were considered statistically significant.

## 3. Results

The mean age of participants was 39.37 years. In the study group (*n* = 91), 34% (*n* = 31) were hypertensives and 66% (*n* = 60) were normotensives (control group). The parameters determined in the polysomnography examination are included in Table 2. The mean BEI of the participants with hypertension was 4.47 ± 2.55, while that of the control group was 2.03 ± 1.24, which indicated a statistically significant difference (*p* < 0.001). Furthermore, statistically significant differences were observed in total, phasic, tonic, and mixed bruxism episodes (Table 3).

The studied groups also showed statistically significant differences in polysomnographic sleep indexes. The hypertensive group was characterized by a contrasting sleep architecture, with significant differences observed in the N2 sleep phase (Table 4); however, no statistically significant difference was observed in other sleep phases. Arousal parameters also differed significantly between the studied groups (*p* = 0.004). The mean AHI of hypertensive participants was 4.77 ± 2.85, while that of the control group was 2.01 ± 1.62 (*p* < 0.001).

**Figure 2:**
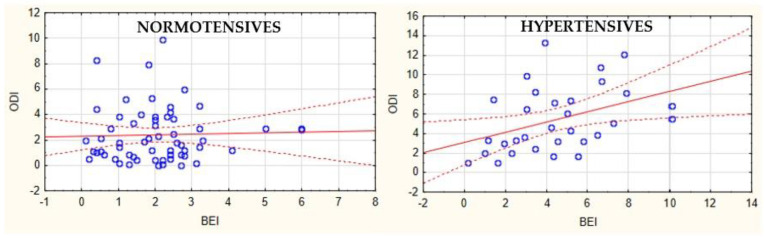
Correlation between ODI and BEI among hypertensives (r = 0.37, *p <* 0.05) and normotensives (r = 0.03, *p >* 0.05). ODI, oxygen desaturation index; BEI, bruxism episode index.

An interesting correlation was found between oxygen desaturation index (ODI) and BEI in both studied groups. In the hypertensive group, this correlation was statistically significant (r = 0.37, *p* < 0.05), whereas it was not statistically significant in the case of normotensives (r = 0.03, *p* > 0.05) (Figure 2).

Furthermore, the studied group showed differences in mean oxygen saturation (SpO_2_), minimal SpO_2_, and % of sleep with saturation of oxygen (SatO_2_) < 90%. All these parameters were found to be decreased in hypertensives compared to normotensives, while ODI was higher in hypertensives. Interestingly, no statistically significant difference in mean heart rate (normotensives: 63.41 ± 10.13 bpm, hypertensives: 61.81 ± 14.00 bpm, *p* = 0.53) was noted between the two groups (Table 4).

Moreover, significant differences were observed in snoring parameters between the studied groups (Table 5). The total snoring percentage was higher in the hypertensive group compared to normotensives regardless of the body position (supine vs. nonsupine) and sleep phase (rapid eye movement (REM) vs. nonrapid eye movement (NREM)).

## 4. Discussion

To our best knowledge, this study is the first to use polysomnography, which is a gold standard in the diagnostics of SB, to analyze a large number (*n* = 91) of hypertensive patients in comparison to normotensives. The study excluded patients with diagnosed OSA, which may influence sleep structure as well as blood pressure. We found that BEI was higher among patients with diagnosed hypertension compared to normotensives. This may be due to the fact that both SB and hypertension share a common pathomechanism, i.e., activation of sympathetic activity and increase in inflammation factors, as previously discovered by Michalek-Zrąbkowska et al. [24]. The study by Michalek-Zrąbkowska et al. proved the positive correlation between inflammatory parameters (C-reactive protein and fibrinogen) and BEI, which may support the hypothesis that SB is linked with cardiovascular risk. Keskinruzgar et al. also confirmed the prevalence of inflammation among patients with SB [25]. These authors measured the inflammation indexes during optical coherence tomography and observed the occurrence of neurodegenerative and inflammatory processes in the examined group.

In an earlier study, Nashed et al. examined 10 patients with SB and 9 patients without SB and investigated the changes in blood pressure [26]. Their study revealed similar findings that patients with SB had more fluctuations in blood pressure as well as more arousals. In addition, increased SB intensity was observed in association with altered sleep architecture. Our previous study also demonstrated that young adults with bruxism had increased blood pressure variability, which is the main risk factor for arterial hypertension [11].

On the other hand, hypertension, as well as higher body mass index, lower mean SpO_2_, and higher SpO_2_ < 90%, constitutes an independent risk factor for increased BEI [18]. Martynowicz et al., for the first time, found that patients with hypertension experienced more bruxism episodes. It is worth mentioning that the authors performed respiratory polygraphy (a method that does not involve video recording of the sleeping patient and is not used to diagnose SB) instead of polysomnography, unlike our study. Moreover, sleep apnea was not excluded.

In summary, the correlation between arterial hypertension and SB remains unclear. Further research is needed to understand whether hypertension is an outcome of SB or if SB leads to the development of hypertension. It can be assumed that age plays a role. Among young adults, bruxism episodes may cause changes in blood pressure, which may predispose to the development of arterial hypertension, as indicated by Nashed et al. and Michalek-Zrabkowska et al. [11,19]. On the other hand, in the case of elderly patients with diagnosed arterial hypertension, systemic inflammation and increased arousal index (ArI) may intensify SB, as shown in the present study and the study by Martynowicz et al. [18]. However, this hypothesis requires further investigation.

Moreover, as far as we know, the prevalence of SB decreases with age; therefore, research should be performed on an older hypertensive group with increased BEI to understand the features and consequences of SB events [27,28].

Patients with OSA were excluded from the present study because OSA is a risk factor for SB and may have influenced the results of examination [29,30]. It has been proven by Konecny et al. that OSA often coexists with hypertension [31]. Furthermore, some studies from the last decade investigated the role of OSA in the development of arterial hypertension [32,33,34]. Nevertheless, the mechanisms remain unclear [35].

One of the most important findings of the present study is increased ArI in hypertensives compared to controls. Recently, Zhao et al. also showed an association between ArI and the prevalence of hypertension [36].

Studies have indicated that increased heart rate is associated with microarousals which in turn are accompanied by increased sympathetic activity and decreased parasympathetic activity [37]. Microarousals affect the heart and brain, resulting in an increase in heart rate and the activation of masseter or temporalis muscles leading to bruxism [38]. Marthol et al. compared heart rate among patients with bruxism and concluded that heart rate alterations may be related to increased heart sympathetic activity [39]. A study performed on a group of 14 patients also proved that a bruxism episode is preceded by a clear increase in heart rate by approximately > 10% of the normal [40]. Thus, in patients with hypertension, increased ArI may induce bruxism episodes.

It is important to link SB with hypertension to ensure an early diagnosis and optimal treatment to prevent the long-term health consequences of cardiac events [41]. Patients with SB present with a higher ArI, which results in sleep fragmentation [13]. Fragmented sleep is less efficient than consolidated sleep and can often result in daytime sleepiness and lack of concentration [42]. More arousals are associated with longer intrasleep wakefulness, lesser sleep efficiency, and greater sleep phase changes in sleep bruxers, as indicated by Trindade and Rodriguez [43]. Increased SB intensity was also found to be associated with increased REM [26]. Ineffective sleep may lead to the development of hypertension as well as increase cardiovascular risk [44].

Moreover, symptoms presented by patients with hypertension such as headaches and tinnitus could indeed be symptoms of SB [28,45,46].

Early examination of SB is critical, as it is classified as a sleeping disorder accompanied by elevated inflammatory parameters [24], which could affect a patient’s homeostasis in various ways. Systemic inflammation is strongly linked with endothelial dysfunction and atherosclerosis, which can lead to hypertension [33]. In this study, we also compared snoring and saturation parameters in the two examined groups and found that hypertensives had decreased SpO_2_ and increased snoring. Oxygen desaturation plays a controversial role in the development of SB [9,47]. Suzuki showed that the mild and brief oxygen fluctuations before the onset of RMMA may slightly influence the development of SB [48]. Nevertheless, only a few studies have confirmed the association between hypoxia and bruxism [18,47]. The present study showed a correlation between BEI and ODI in hypertensives, but not in normotensives. This may indicate the different pathomechanism of SB in hypertensives and explains the contradictory results observed in previous studies.

The oxygen desaturation index was indeed higher among the hypertensives group, and that can be explained by the fact that hypertensive patients have also other causes of presenting the desaturation, such as increased number of respiratory events or higher body mass index. Taking the heart rate into consideration, there are many more other compounds affecting this parameter such as increased sympathetic activity, age, other medical conditions, medications, diet, fitness level, and psychological factors.

Increased snoring can be explained by the fact that the obstructive process begins to occur in respiratory airways in patients with hypertension. In addition, hypertension is associated with altered metabolic pathways, which may result in overweight and consequently obstruction in the upper airways leading to lower SpO_2_ and more snoring episodes throughout the night. Further research is needed to study the pathophysiology of bruxism in patients with arterial hypertension together with an analysis of upper airway obstruction.

In this study, a limitation was the fact that the patients did not perform automatic blood pressure measurement. It would be interesting to analyze the correlations between arterial blood pressure parameters and SB; however, it was beyond the scope of this study.

What is more, the study group representing hypertensive patients consisted of 14 men and 17 women, whereas the control group was mostly represented by women, and this is an evident limitation of this research. Additionally, BMI was not evaluated in both groups, although it plays a very important role as a risk factor for high blood pressure and limits the research. We have not assessed temporomandibular issues in the study either.

A limitation of the study was that patients spent only one night in the Sleep Laboratory for the polysomnographic examination. Examination for two or more nights would have given us more reliable information. An adaptive night is indeed very important, but due to the state of Polish healthcare, a longer stay for the research was unfortunately not possible.

## 5. Conclusions

1. Nonapneic hypertensives had higher SB intensity, altered sleep architecture, increased snoring, and decreased mean oxygen saturation compared to normotensives.
2. The association between oxygen desaturations and bruxism episodes exists only in hypertensives, but not in normotensives.
3. Dental screening is necessary for patients with arterial hypertension, especially those presenting with the symptoms of SB.

## Figures and Tables

**Table 1:** Characteristics of the study group (*n* = 91).

	Hypertensives (*n* = 31)	Normotensives (*n* = 60)	Total
Female	17	46	63
Male	14	14	28
Age ± SD	48.06 ± 14.76	34.88 ± 11.24	39.37 ± 13.96

SD, standard deviation.

**Table 2:** Polysomnographic parameters determined in the study group (*n* = 91).

Parameter	Mean	Parameter	Mean
BEI (/h)	2.86 ± 2.13	SL (min)	21.93 ± 22.93
Phasic BEI (/h)	1.62 ± 3.77	AHI (/h)	2.95 ± 2.48
Tonic BEI (/h)	1.08 ± 1.20	ODI (/h)	3.42 ± 2.96
Mixed BEI (/h)	0.66 ± 0.99	Mean SpO_2_ (%)	94.22 ± 1.66
WASO (min)	38.71 ± 38.25	Mean heart rate (/min)	62.87 ± 11.55
N1 (%)	3.65 ± 4.57	Snore supine (% TST)	12.90 ± 19.97
N2 (%)	49.18 ± 9.43	Snore nonsupine (% TST)	7.46 ± 14.88
N3 (%)	23.96 ± 7.94	REM Snore (% TST)	5.22 ± 9.90
REM (%)	23.18 ± 6.41	NREM Snore (% TST)	11.49 ± 16.67

BEI, bruxism episode index; WASO, wake after sleep onset; N1, N2, N3, NREM stages 1, 2, 3; REM, rapid eye movement; SL, sleep latency; AHI, apnea–hypopnea index; ODI, oxygen desaturation index; SpO_2_, oxygen saturation; NREM, nonrapid eye movement.

**Table 3:** Bruxism episode index in the study group and control group.

Bruxism Episode Index (/h)	Hypertensives *n* = 31	Normotensives *n* = 60	*p*-Value
Mean	4.47 ± 2.55	2.04 ± 1.24	**<0.001**
Phasic	3.23 ± 6.09	0.79 ± 085	**<0.01**
Tonic	1.69 ± 1.75	0.77 ± 0.59	**<0.001**
Mixed	1.08 ± 1.52	0.45 ± 0.43	**<0.01**

**Table 4:** Polysomnographic parameters determined in the study group and control group.

	Hypertensives *n* = 31	Normotensives *n* = 60	*p*-Value
N1 (%TST)	3.98 ± 3.50	3.48 ± 5.06	>0.05
N2 (%TST)	46.48 ± 10.69	50.57 ± 8.47	**<0.001**
N3 (%TST)	24.96 ± 9.11	23.44 ± 7.28	>0.05
R (%TST)	24.54 ± 6.91	22.48 ± 6.08	>0.05
Arousals (/h)	4.35 ± 3.29	2.79 ± 1.86	**<0.01**
AHI (/h)	4.77 ± 2.85	2.01 ± 1.62	**<0.001**
ODI (/h)	5.42 ± 3.32	2.39 ± 2.12	**<0.001**
Mean SpO_2_ (%)	93.25 ± 1.87	94.71 ± 1.31	**<0.001**
Minimal SpO_2_ (%)	84.26 ± 8.14	90.15 ± 3.96	**<0.001**
SpO_2_ < 90%	6.20 ± 12.38	0.78 ± 4.08	**<0.01**
Average desaturation drop (%)	3.51 ± 0.53	3.12 ± 1.08	>0.05
HR (/min)	61.82 ± 14.00	63.42 ± 10.14	>0.05
HR maximum (/min)	100.44 ± 34.65	81.75 ± 55.51	>0.05
HR minimum (/min)	50.60 ± 9.60	50.47 ± 7.94	>0.05

N1, N2, N3, nonrapid eye movement stages 1, 2, 3; R, nonrapid eye movement phase of sleep; TST, total sleep time; AHI, apnea–hypopnea index; ODI, oxygen desaturation index; SpO_2_, oxygen saturation; HR, heart rate. Statistically significant values are shown in bold (*p* < 0.05).

**Table 5:** Snoring parameters (% TST) determined in the study group and control group.

	Hypertensives *n* = 31	Normotensives *n* = 60	*p*-Value
Supine snore	24.04 ± 23.28	7.14 ± 15.29	**<0.001**
Nonsupine snore	15.48 ± 19.78	3.32 ± 9.35	**<0.001**
REM snore	10.61 ± 13.94	2.43 ± 5.24	**<0.001**
NREM snore	22.15 ± 19.62	5.99 ± 11.72	**<0.001**
Total snore	18.80 ± 17.67	5.25 ± 10.03	**<0.001**

REM, rapid eye movement; NREM, nonrapid eye movement; TST, total sleep time. Statistically significant values are shown in bold.

## Data Availability

The data that support the findings of this study are available on request from the corresponding author and are not publicly available due to privacy or ethical restrictions.
